# CircMTO1 Attenuated Acute Kidney Injury Through Regulating miR-337

**DOI:** 10.1007/s10753-020-01209-w

**Published:** 2020-03-10

**Authors:** Chuan-chuan Shi, Lu-yan Pan, Zhi-yong Peng, Jian-guo Li

**Affiliations:** 1 Department of Intensive Care Unit, Zhongnan Hospital of Wuhan University, Wuhan, 430071 Hubei China; 2 Henan Health Cadre College, Zhengzhou, 450000 Henan China

**Keywords:** sepsis, AKI, circMTO1, miR-337, KLF6

## Abstract

Acute kidney injury (AKI) is an independent risk factor for the increased risk of death in patients with sepsis. In the current study, we first investigated the expression of circMTO1 in sepsis-induced AKI, and the underlying mechanism was further elucidated. The results showed that circMTO1 expression level was significantly decreased in serums and kidney tissues of US rats and RMCs treated with LPS. Besides, circMTO1 overexpression promoted cell viability, suppressed cell apoptosis and cytokines production of LPS-treated RMCs. Bioinformatics analysis showed that circMTO1 served as a sponge for miR-337. Furthermore, circMTO1 could inhibit the expression of KLF6. Altogether, our study first reported that circMTO1 expression was decreased in sepsis-induced AKI rat models and RMCs treated with LPS. CircMTO1 overexpression could attenuate AKI development by sponging miR-337 and regulating KLF6 expression, which may provide new ideas for evaluation the pathogenesis and the treatment of sepsis-induced AKI.

## INTRODUCTION

Sepsis is a systemic inflammatory response syndrome caused by pathogenic microorganisms invading the body, which can give rise to septic shock and multiple organ dysfunction syndromes [1]. Sepsis is a common disease in critically ill patients, with a 20–30% in-hospital mortality 63 rate [2]. Kidney is one of the most sensitive organs which can be involved in sepsis. And acute kidney injury (AKI) is an independent predictor of mortality of patients with sepsis [3, 4]. Despite extensive advancements in sepsis-induced AKI therapeutic strategies, the prognosis of patients still remains unfavorable [5]. Thus, it is imperative to explore the molecular mechanisms underlying sepsis-induced AKI.

Compared with regular linear RNAs, circRNAs (circRNAs) exhibit the notable characteristic of covalently closed loop structures with neither 5′ caps nor 3′ polyadenylated tails, leading to a stable structure and high tissue-specific expression [6, 7]. Abnormal circRNAs expression has been detected in the development of several diseases, such as atherosclerosis [8], myocardial infarction [9], and nervous system disorders [10]. Until now, few studies focused on the role of circRNAs in the progression of sepsis-induced AKI. CircMTO1 (mitochondrial translation optimization 1 homolog, circ_0007874) has been demonstrated to play an inhibitory role in some kinds of tumor development. However, the molecular function of circMTO1 in sepsis-induced AKI was still unclear.

MicroRNAs (miRNAs) belong to noncoding single-stranded RNAs which are involved in many physiological processes like cell proliferation, apoptosis, and migration [11]. Previous studies have reported the association between microRNAs and AKI [12]. Nevertheless, the role of miR-337 in AKI was not yet elucidated. In the present study, we first investigated the effect of circMTO1 on the progression of sepsis-induced AKI, and the underlying mechanism was further elucidated.

## MATERIALS AND METHODS

### Establishment of the US Model

Adult male Sprague-Dawley (SD) rats weighing 230 ± 10 g were obtained from Beijing Laboratory Animal Research Center (Beijing, China). The rats were housed under standard environmental conditions (temperature 25 ± 2 °C, humidity 55 ± 5%, and 12-h light/12-h dark cycle) and given free access to food and tap water. All rats were randomly divided two groups with 6 in each group and then anesthetized *via* intraperitoneal injection of chloral hydrate (10%, 0.3 ml/kg). The rats were fixed in a supine position, and the incision was then made along the left rectus muscle. In urine-derived sepsis (US) group, the left ureter is incised and ligated in the middle section, resulting in acute upper urinary obstruction. An *Escherichia coli* suspension (ATCC 25922, 1 × 10^8^/ml, 0.5 ml/kg) was then injected into the distal ureter as previously described [13]. In the sham group, the left ureter was only separated and the incision with sutured. All rats were sacrificed 24 h after the surgery. The 5 ml serum samples from these rats were centrifuged at 12 000*g* for 3 min, and the supernatant was frozen at liquid nitrogen, and the harvested left kidney tissues were stored at liquid nitrogen for further analysis. This study was carried out with the approval of the Institutional Animal Care and Use Committee of Tianjin Medical University.

### Cell Culture and Transfection

Rat mesangial cells (RMCs, purchased from the Cell Bank of the Chinese Academy) were maintained in Dulbecco’s modified Eagle’s medium containing 10% fetal bovine serum and 1% penicillin/streptomycin under an atmosphere of a humidified air and 5% CO_2_ at 37 °C. Until around 60% of confluence, RMCs cells were treated with 100 ng/mL LPS for 24 h to induce injury inflammation. Cells were seeded in 6-well plates and then transfected with si-circMTO1, miR-337 inhibitor, or their corresponding controls mixed with lipofectamine 2000 reagent according to the manufacturer’s protocols.

### Cell Viability

RMCs (1 × 10^3^ cells/well) with different transfections were seeded in 96-well plates and cultured for 0, 24, 48, 72, 96 h. After being washed three times with PBS, cells were treated with 500 μg/mL of MTT solution for 3 h. Subsequently, 200 μl of dimethylsulfoxide (DMSO) was added to dissolve precipitates. The optical density (OD) of each well was measured at 490 nm under a microplate spectrophotometer.

### Cell Apoptosis

The cells were suspended in the binding buffer, fixed in ice-cold 70% ethanol, and stained with FITC/Annexin V and PI in the dark at room temperature for 15 min. The apoptosis detection was performed by flow cytometry assay (FACScan, BD Biosciences, San Jose, CA, USA) and analyzed using CellQuest software.

### Enzyme Linked Immunosorbent Assay

According to the manufacturer’s instruction, the concentration of inflammatory cytokines in serum (TNF-α, IL-1β and IL-6) was determined using an ELISA Kit. Blood urea nitrogen (BUN) and serum creatinine were determined using a Urea Nitrogen Colorimetric Detection Kit and Creatinine Urinary Detection Kit (Invitrogen, CA, USA).

### Real Time Quantitative PCR Analysis

Total RNA from kidney tissues and RMCs was isolated using TRIzol reagent (Invitrogen) following the manufacturer’s protocol. And cDNA synthesis was conducted using the TaqMan MicroRNA Reverse Transcription Kit (Applied Biosystems) for miR-337 and using One Step PrimeScript cDNA Kit (Qiagen) for circMTO1 and KLF6. RT-PCR was performed using the SYBR® Premix Ex Taq™ II (Takara) and the Applied Biosystems 7500 Real-time PCR System (Applied Biosystem). The relative expression was analyze by using 2-ΔΔCT method and normalized to GAPDH or U6. The primers were as follows: circMTO1 forward 5′- TTACCAGCCGAGTAGAGTTCC-3′ and reverse 5′- ATCCATTCCTTCAGGTTCCAAC-3′; miR-630 forward 5′- CGCTTCAGCTCCTATATGA-3′ and reverse 5′- GTGCAGGGTCCGAGGT-3′; lnc-NC 5’-UGGACAACAUGGGCUCU-3′; KLF6 forward 5’-TCAAATGCTATCCCCTTTCC-3′ and reverse 5′- CCAGGGCTAGGAAGTAGGAG-3′; U6 forward 5’-GCTCGCTTCGGCAGCACA-3′ and reverse 5′-GAGGTA TTCGCA CCAGAG GA-3′; and GAPDH forward 5′- TGTGGGCATCAATGGATTTGG-3′ and reverse 5′- ACACCATGTATTCCGGGTCAAT-3′.

### Western Blotting Analysis

Proteins were extracted from kidney tissues and cultured RMCs by RIPA buffer (Sigma-Aldrich; Merck KGaA) containing a mixture of protease inhibitors. Equal quantities of protein were separated *via* SDS-PAGE on a 10% gel and transferred to PVDF membranes. After blocking with 5% skimmed milk, the membrane was incubated with the primary antibodies anti-KLF6 at 4 °C overnight, followed by incubating with anti-rabbit horseradish peroxidase-conjugated secondary antibody and detected using enhanced chemiluminescence detection system.

### Luciferase Reporter Assay

The fragment of circMTO1 obtaining wild type (WT) or Mutant (Mut) miR-337 binding sites was constructed by Genomeditech (Shanghai, China) and inserted into pGL3 Basic vector. Cells were seeded into 24-well plates and co-transfected with the luciferase reporter constructs, miR-337 inhibitor, and Renilla luciferase construct (Promega) and incubated for 24 h. The relative luciferase activities were measured using the Dual-Luciferase Reporter System (Promega).

### Statistical Analysis

All measurement data were expressed as the mean ± standard deviation. Differences were calculated with Student’s t test or one-way ANOVA. All statistical analyses were performed using the SPSS 17.0 software and GraphPad Prism 6. A value of *P* < 0.05 was considered to indicate a statistically significant difference.

## RESULTS

### CircMTO1 Expression Was Decreased in Serum and Kidney Tissue of US Rats

**Fig. 1:**
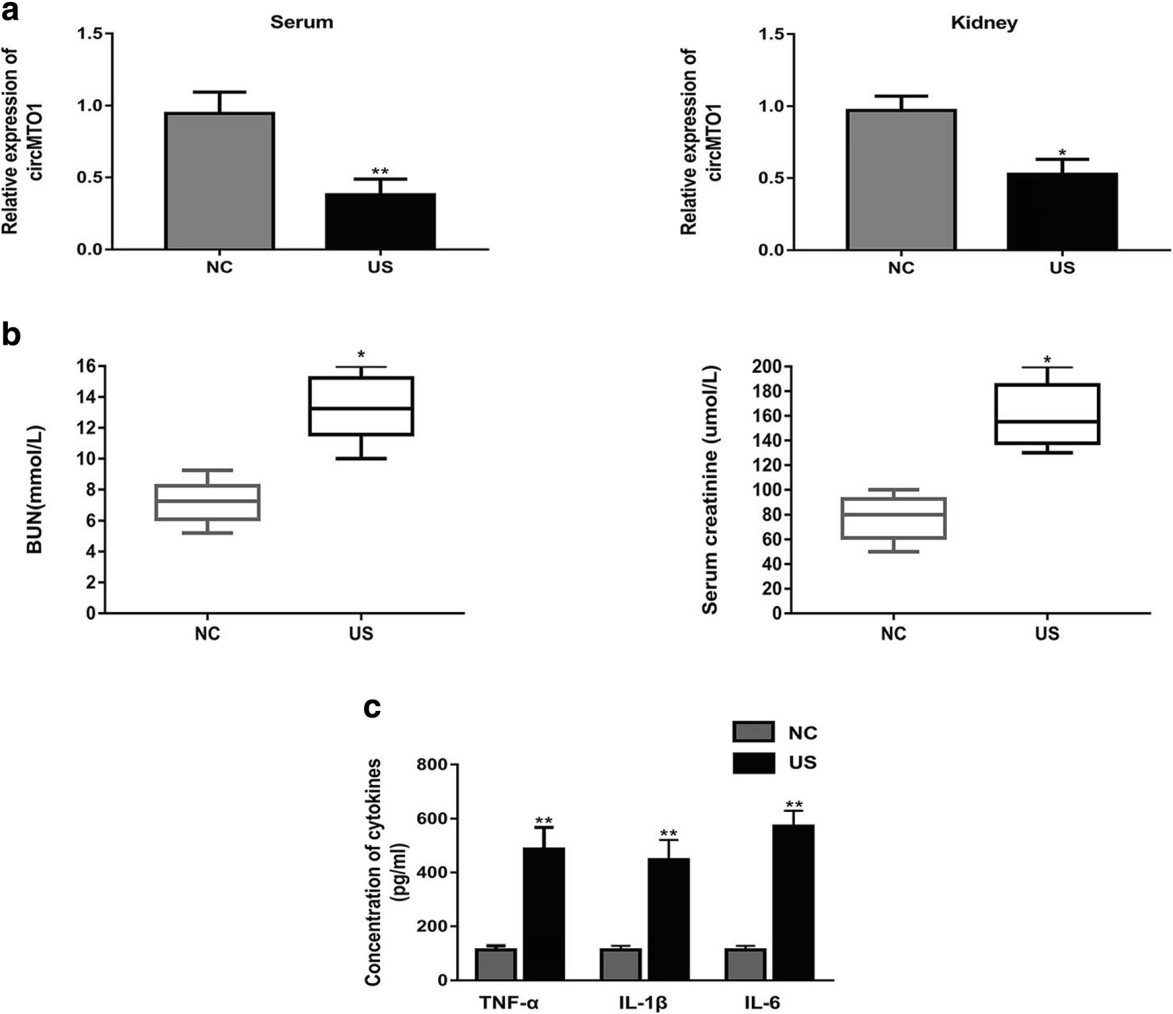
CircMTO1 expression was decreased in serum and kidney tissue of US rats. **a** circMTO1 expression level was significantly downregulated in serums and kidney tissues of US rats in comparison with that in control rats. **b** BUN and serum creatinine) were significantly upregulated in US rats. **c** The concentration of inflammation cytokines (TNF-α, IL-1β, and IL-6) was significantly higher in US rats than those in control rats. ^*^P < 0.05, ^**^P < 0.01 compared to control group.

To evaluate the role of circMTO1 in sepsis-AKI, we first measured circMTO1 expression in serum and kidney tissues of US rats using RT-qPCR. The result showed that circMTO1 expression level was significantly downregulated in serums and kidney tissues of US rats in comparison with that in control rats (Fig. 1a). As expected, renal function indicators (BUN and serum creatinine) were upregulated in US rats (Fig. 1b). Besides, the concentration of inflammation cytokines (TNF-α, IL-1β and IL-6) was significantly higher in US rats than those in control rats (Fig. 1c).

### CircMTO1 Overexpression Promoted Cell Viability, Suppressed Cell Apoptosis, and Cytokines Production of LPS-Treated RMCs

**Fig. 2:**
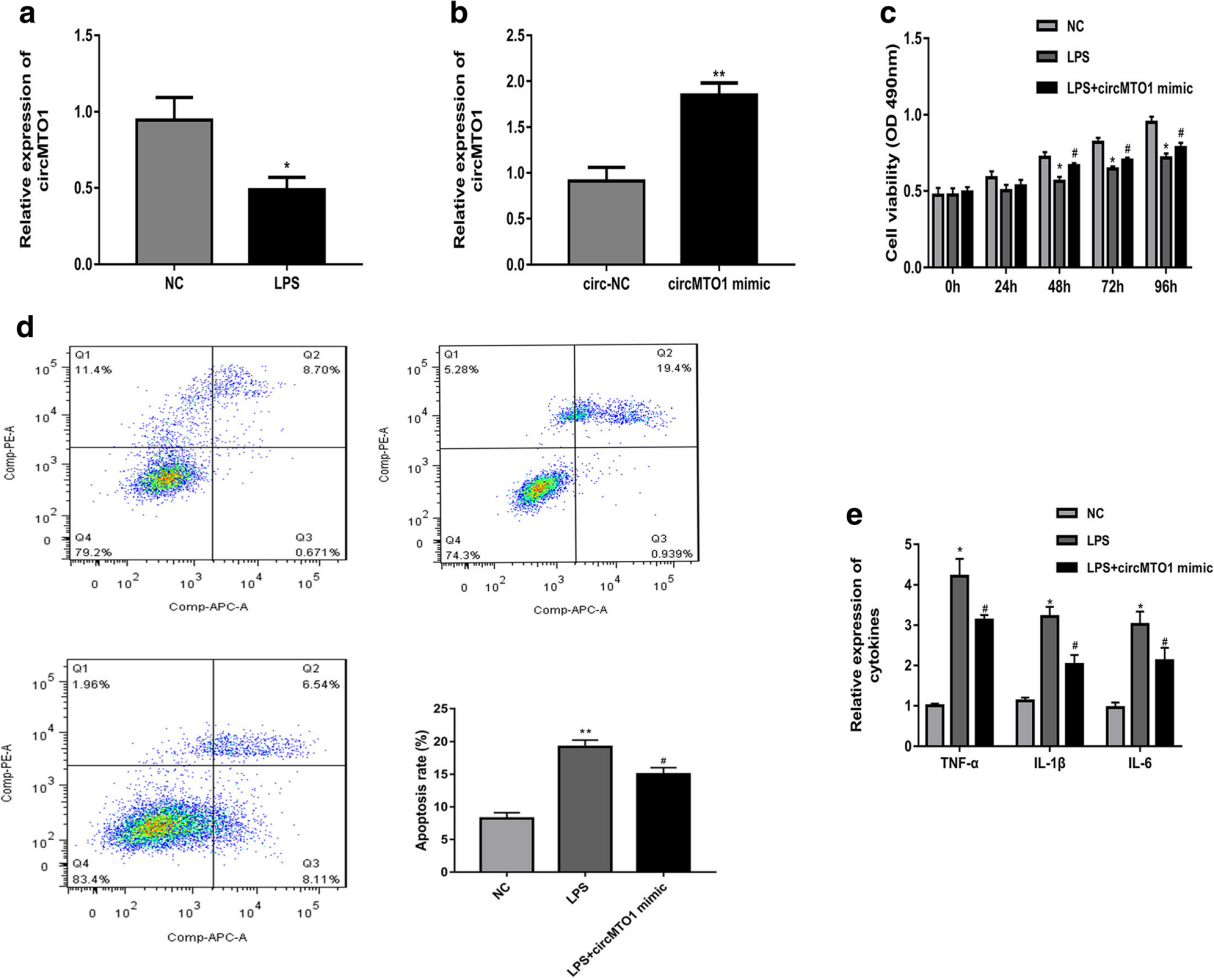
CircMTO1 overexpression promoted cell viability, suppressed cell apoptosis and cytokines production of LPS-treated RMCs. **a**: The expression of circMTO1 was notably decreased in RMCs treated with LPS**. b** circMTO1 overexpression was established in LPS-treated RMCs by transfection. **c** MTT assays revealed circMTO1 mimic accelerated the proliferation of LPS-treated RMCs significantly. **d** Flow cytometry revealed that cell apoptosis rate was lower in LPS-treated RMCs after transfection with circMTO1 mimic. **e**: circMTO1 overexpression could obviously downregulated the expression of related cytokines. ^*^P < 0.05, ^**^P < 0.01 compared to control group; ^#^*P* < 0.05 compared to LPS group.

Next we explored the role of circMTO1 in LPS-treated RMCs. After treated with LPS, the expression of circMTO1 was notably decreased in RMCs (Fig. 2a). And then circMTO1 overexpression was established in LPS-treated RMCs by transfection (Fig. 2b). MTT assays revealed that circMTO1 mimic accelerated the proliferation of LPS-treated RMCs significantly (Fig. 2c). And flow cytometry analysis demonstrated that cell apoptosis rate was lower in LPS-treated RMCs after transfection with circMTO1 mimic (Fig. 2d). In addition, circMTO1 overexpression could obviously downregulated the expression of related cytokines (Fig. 2e).

### CircMTO1 Serves as a Sponge for miR-337

**Fig. 3:**
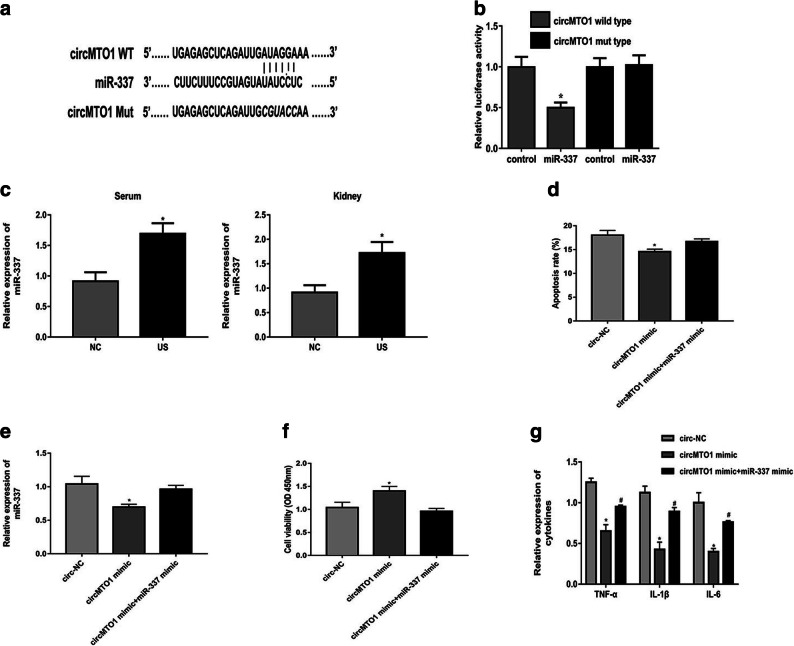
CircMTO1 serves as a sponge for miR-337. **a** miR-337 was observed to have a potential binding site with circMTO1. **b** miR-337 mimic suppressed the luciferase activity of only circMTO1 wild type in cells. **c** miR-337 expression was significantly upregulated in US rat models and LPS-treated RMCs. **d** circMTO1 overexpression could downregulated miR-337 level significantly in RMCs, which was reversed by miR-337 mimic. **e** MTT assay demonstrated that miR-337 mimic could repress the induction by circMTO1 overexpression in the proliferation of RMCs treated with LPS at 96 h. **f** miR-337 mimic could increase the apoptosis rate of LPS-treated RMCs transfected with circMTO1 mimic. **g** miR-337 mimic obviously promoted cytokines production in cells transfected with circMTO1 mimic. ^*^P < 0.05 compared to control group; ^#^*P* < 0.05 compared to circMTO1 mimic group.

Given that circRNA has been shown to act as miRNA sponge [14], we further predicted the potential circRNA-miRNA interactions using CircInteractome (https://circinteractome.nia.nih.gov/). As shown in Fig. 3 a, miR-337 was observed to have a potential binding site with circMTO1. Luciferase reporter assay verified that miR-337 mimic suppressed the luciferase activity of only circMTO1 wild type in cells (Fig. 3b). And miR-337 expression was significantly upregulated in US rat models and LPS-treated RMCs (Fig. 3c). Under LPS-stimulated condition, circMTO1 overexpression could downregulated miR-337 level significantly in RMCs, which was reversed by miR-337 mimic (Fig. 3d). MTT assay demonstrated that miR-337 mimic could repress the induction by circMTO1 overexpression in the proliferation of RMCs treated with LPS at 96 h (Fig. 3e). Besides, miR-337 mimic could increase the apoptosis rate of LPS-treated RMCs transfected with circMTO1 mimic (Fig. 3f). As expected, miR-337 mimic obviously promoted cytokines production in cells transfected with circMTO1 mimic (Fig. 3g).

### CircMTO1 Overexpression Could Inhibit the Expression of KLF6

**Fig. 4:**
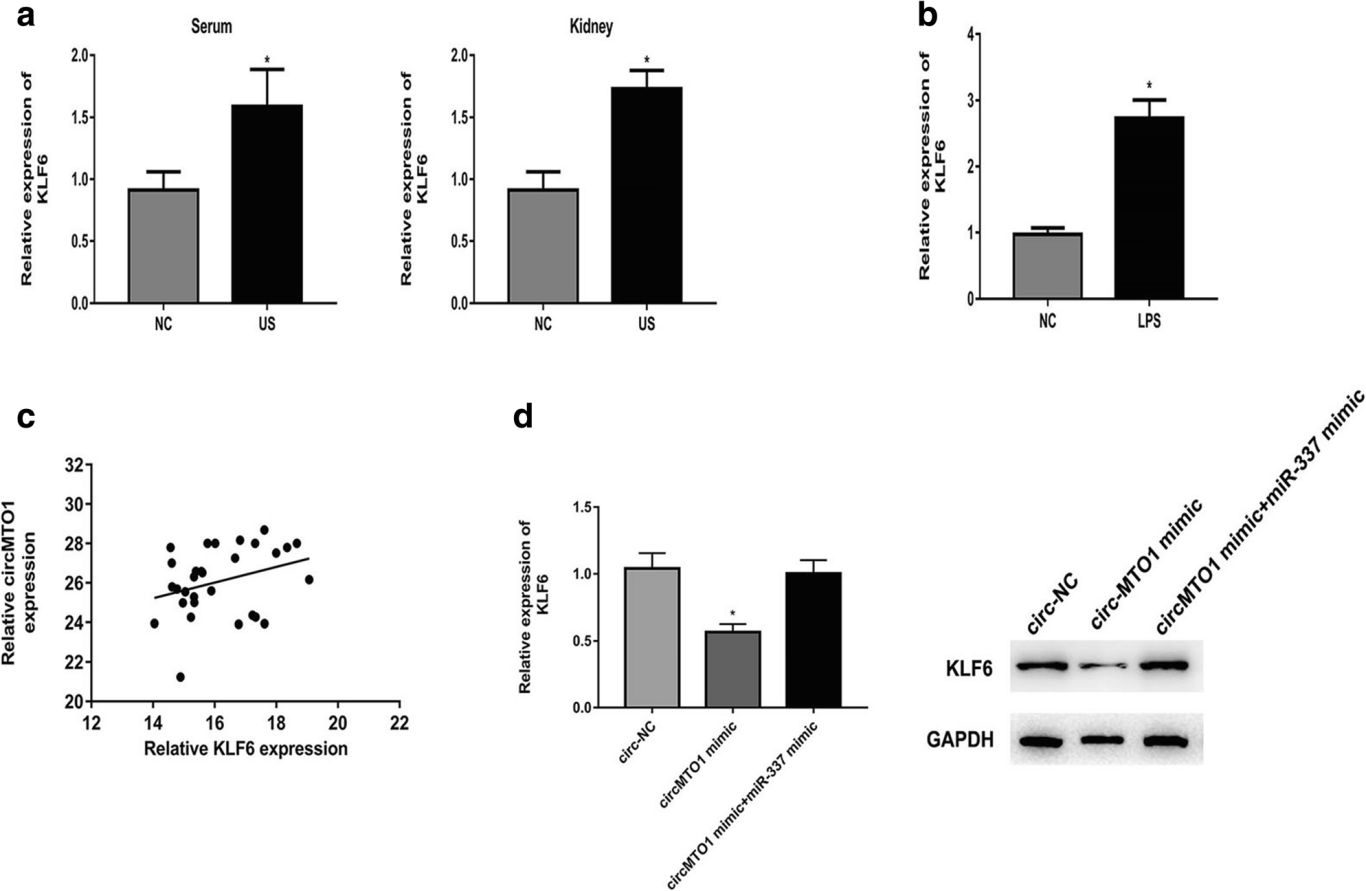
CircMTO1 overexpression could inhibit the expression of KLF6. **a** KLF6 expression level was significantly upregulated in US rats’ models. **b** KLF6 expression level was significantly upregulated in RMCs treated with LPS. **c** KLF6 expression was positively correlated with circMTO1 expression in kidney tissues. **d** circMTO1 overexpression could suppress the expression of KLF6 obviously, which was reversed by miR-337 mimic at both mRNA and protein levels. ^*^P < 0.05 compared to control group.

KLF6 has been confirmed to have a potential role in inflammatory response [15]. Subsequently, we analyzed the effect of circMTO1 on the expression of KLF6 in LPS-treated RMCs. The result showed that KLF6 expression level was significantly upregulated in US rat models and RMCs treated with LPS (Fig. 4a, b). Meanwhile, KLF6 expression was positively correlated with circMTO1 expression in kidney tissues (Fig. 4c). Further, circMTO1 overexpression could suppress the expression of KLF6 obviously, which was reversed by miR-337 mimic (Fig. 4d).

## DISCUSSION

Up to now, circRNA have been reported to participate in many crucial biological events such as cell proliferation, apoptosis, metastasis, and differentiation [16, 17]. A growing number of studies have shown that aberrant expression of circRNA plays a regulatory role in different diseases *via* different mechanisms [18–20]. In addition, circRNAs may have potential as clinical diagnostic markers and therapeutic targets in sepsis [21, 22]. Here, we first explored the role of circMTO1 in sepsis-AKI model. The results showed circMTO1 expression level was significantly decreased in serums and kidney tissues of US rats. Meanwhile, the concentration of inflammation cytokines (TNF-α, IL-1β, and IL-6) was significantly higher in US rats. And then we observed that circMTO1 overexpression could promote cell viability and inhibited cell apoptosis and cytokines production of LPS-treated RMCs. These data implied circMTO1 mimic could suppress the biological behavior of RMCs under LPS-stimulated condition.

Given that circRNAs could regulate mRNA translation indirectly by competing for miRNAs [23], we predicted that circMTO1 functionally act as the sponge for miR-337 through miRNA screening. Previous research has demonstrated that suppressing miR-337 could attenuate inflammation progression in some diseases [24, 25]. Likewise, miR-337 expression level was significantly upregulated in US rat models and RMCs cells treated with LPS. The recovery experiment revealed that miR-337 mimic could reverse the inhibitory effect on inflammation of circMTO1. Krüppel-like factors (KLFs) are highly conserved zinc-finger proteins that regulate cellular transcription [26]. KLF6 has been reported to participate in proinflammatory gene expression in kidney diseases [27]. We noticed that KLF6 expression was significantly upregulated in US rats and RMCs. Besides, circMTO1 overexpression could suppress the expression of KLF6 obviously, which was reversed by miR-337 mimic. These data suggested that circMTO1 may function through regulation of miR-337/KLF6 axis in AKI-sepsis progression.

## CONCLUSION

Altogether, we first discovered that circMTO1 expression was decreased in sepsis-induced AKI rat models and RMCs treated with LPS. CircMTO1 overexpression could attenuate AKI development by sponging miR-337 and regulating KLF6 expression, which may provide new ideas for the evaluation of the pathogenesis and the treatment of sepsis-induced AKI.

## References

[1] Singer M, Deutschman CS, Seymour CW, Shankar-Hari M, Annane D, Bauer M, Bellomo R, Bernard GR, Chiche JD, Coopersmith CM, Hotchkiss RS, Levy MM, Marshall JC, Martin GS, Opal SM, Rubenfeld GD, van der Poll T, Vincent JL, Angus DC (2016). The third international consensus definitions for sepsis and septic shock (Sepsis-3). JAMA.

[2] Angus DC, Linde-Zwirble WT, Lidicker J, Clermont G, Carcillo J, Pinsky MR (2001). Epidemiology of severe sepsis in the United States: Analysis of incidence, outcome, and associated costs of care. Critical Care Medicine.

[3] Lopes JA, Fernandes P, Jorge S, Resina C, Santos C, Pereira A, Neves J, Antunes F, Gomes da Costa A (2010). Long-term risk of mortality after acute kidney injury in patients with sepsis: A contemporary analysis. BMC Nephrology.

[4] Plataki M, Kashani K, Cabello-Garza J, Maldonado F, Kashyap R, Kor DJ, Gajic O, Cartin-Ceba R (2011). Predictors of acute kidney injury in septic shock patients: An observational cohort study. Clinical Journal of the American Society of Nephrology.

[5] Angus DC (2011). The search for effective therapy for sepsis: Back to the drawing board?. JAMA.

[6] Hentze MW, Preiss T (2013). Circular RNAs: splicing's enigma variations. The EMBO Journal.

[7] Vicens Q, Westhof E (2014). Biogenesis of circular RNAs. Cell.

[8] Holdt LM, Stahringer A, Sass K, Pichler G, Kulak NA, Wilfert W, Kohlmaier A, Herbst A, Northoff BH, Nicolaou A, Gäbel G, Beutner F, Scholz M, Thiery J, Musunuru K, Krohn K, Mann M, Teupser D (2016). Circular non-coding RNA ANRIL modulates ribosomal RNA maturation and atherosclerosis in humans. Nature Communications.

[9] Garikipati VNS, Verma SK, Cheng Z, Liang D, Truongcao MM, Cimini M, Yue Y, Huang G, Wang C, Benedict C, Tang Y, Mallaredy V, Ibetti J, Grisanti L, Schumacher SM, Gao E, Rajan S, Wilusz JE, Goukassian D, Houser SR, Koch WJ, Kishore R (2019). Circular RNA CircFndc3b modulates cardiac repair after myocardial infarction via FUS/VEGF-A axis. Nature Communications.

[10] You X, Vlatkovic I, Babic A, Will T, Epstein I, Tushev G, Akbalik G, Wang M, Glock C, Quedenau C, Wang X, Hou J, Liu H, Sun W, Sambandan S, Chen T, Schuman EM, Chen W (2015). Neural circular RNAs are derived from synaptic genes and regulated by development and plasticity. Nature Neuroscience.

[11] Bartel DP (2004). MicroRNAs: Genomics, biogenesis, mechanism, and function. Cell.

[12] Jones TF, Bekele S, O'Dwyer MJ, Prowle JR (2018). MicroRNAs in acute kidney injury. Nephron.

[13] Shen J, Zhang J, Jiang X, Wang H, Pan G (2018). LncRNA HOX transcript antisense RNA accelerated kidney injury induced by urine-derived sepsis through the miR-22/high mobility group box 1 pathway. Life Sciences.

[14] Hansen TB, Jensen TI, Clausen BH, Bramsen JB, Finsen B, Damgaard CK, Kjems J (2013). Natural RNA circles function as efficient microRNA sponges. Nature.

[15] Date D, Das R, Narla G, Simon DI, Jain MK, Mahabeleshwar GH (2014). Kruppel-like transcription factor 6 regulates inflammatory macrophage polarization. The Journal of Biological Chemistry.

[16] Chen LL, Yang L (2015). Regulation of circRNA biogenesis. RNA Biology.

[17] Lasda E, Parker R (2014). Circular RNAs: Diversity of form and function. RNA.

[18] Du WW, Fang L, Yang W, Wu N, Awan FM, Yang Z, Yang BB (2017). Induction of tumor apoptosis through a circular RNA enhancing Foxo3 activity. Cell Death and Differentiation.

[19] Rybak-Wolf A, Stottmeister C, Glazar P, Jens M, Pino N, Giusti S, Hanan M (2015). Circular RNAs in the mammalian brain are highly abundant, conserved, and dynamically expressed. Molecular Cell.

[20] Wu Y, Zhang Y, Wang JJ (2017). CircRNA hsa_circ_0005105 upregulates NAMPT expression and promotes chondrocyte extracellular matrix degradation by sponging miR-26a. Cell Biology International.

[21] Dai Y, Liang Z, Li Y, Li C, Chen L (2017). Circulating long noncoding RNAs as potential biomarkers of sepsis: A preliminary study. Genetic Testing and Molecular Biomarkers.

[22] Huang Q, Huang C, Luo Y, He F, Zhang R (2018). Circulating lncRNA NEAT1 correlates with increased risk, elevated severity and unfavorable prognosis in sepsis patients. The American Journal of Emergency Medicine.

[23] Han B, Chao J, Yao H (2018). Circular RNA and its mechanisms in disease: From the bench to the clinic. Pharmacology & Therapeutics.

[24] Mar-Aguilar F, Mendoza-Ramirez JA, Malagon-Santiago I, Espino-Silva PK, Santuario-Facio SK, Ruiz-Flores P, Rodriguez-Padilla C, Resendez-Perez D (2013). Serum circulating microRNA profiling for identification of potential breast cancer biomarkers. Disease Markers.

[25] Xia P, Gao X, Duan L, Zhang W, Sun YF (2018). Mulberrin (Mul) reduces spinal cord injury (SCI)-induced apoptosis, inflammation and oxidative stress in rats via miroRNA-337 by targeting Nrf-2. Biomedicine & Pharmacotherapy.

[26] Kaczynski J, Cook T, Urrutia R (2003). Sp1- and Kruppel-like transcription factors. Genome Biology.

[27] Rane MJ, Zhao Y, Cai L (2019). Krupsilonppel-like factors (KLFs) in renal physiology and disease. EBioMedicine.

